# Mechanical Pressure Characterization of CNT-Graphene Composite Material

**DOI:** 10.3390/mi11111000

**Published:** 2020-11-12

**Authors:** Asar Ali, Farman Ali, Muhammad Irfan, Fazal Muhammad, Adam Glowacz, Jose Alfonso Antonino-Daviu, Wahyu Caesarendra, Salman Qamar

**Affiliations:** 1 Department of Electrical Engineering, Wah Engineering College, University of Wah, Rawalpindi 47040, Pakistan; 2 Department of Electrical Engineering, Qurtuba University of Science and IT, Dera Ismail Khan, Khyber Pakhtunkhwa 29050, Pakistan; mskhan131@qurtuba.edu.pk; 3 Electrical Engineering Department, College of Engineering, Najran University Saudia Arabia, Najran 61441, Saudi Arabia; miditta@nu.edu.sa; 4 Electrical Engineering Department, University of Engineering Technology, Mardan 23200, Pakistan; 5 Department of Automatic, Control and Robotics, AGH University of Science and Technology, 30-059 Krakow, Poland; 6 Instituto Tecnologico de la Energía Camino de Vera s/n, Universitat Politecnica de Valencia, 46022 Valencia, Spain; joanda@die.upv.es; 7 Faculty of Integrated Technologies, Universiti Brunei Darussalam, Jalan Tungku Link, Gadong BE1410, Brunei; caesarendra@ubd.edu.bn

**Keywords:** carbon nanotubes, graphene, pressure sample, resistance, composite, pressure

## Abstract

Carbon nanotubes (CNTs) and graphene are extensively studied materials in the field of sensing technology and other electronic devices due to their better functional and structural properties. Additionally, more attention is given to utilize these materials as a filler to reinforce the properties of other materials. However, the role of weight percentage of CNTs in the piezoresistive properties of these materials has not been reported yet. In this work, CNT-graphene composite-based piezoresistive pressure samples in the form of pellets with different weight percentages of CNTs were fabricated and characterized. All the samples exhibit a decrease in the direct current (DC) resistance with the increase in external uniaxial applied pressure from 0 to 74.8 kNm^−2^. However, under the same external uniaxial applied pressure, the DC resistance exhibit more decrease as the weight percentage of the CNTs increase in the composites.

## 1. Introduction

Graphene is one atom thick two-dimensional honeycomb layers sp^2^-hybredized carbon atoms; while multi-walled carbon nanotubes (MWCNTs) are formed by the rolling the graphene sheets into concentric cylindrical shapes with interlayer separation of 0.34 nm [1,2]. More than one hundred years back, it has been invented that carbon nanomaterials are sensitive to the variation in environmental pressure, and Alexander Graham Bell patented the first telephone in 19th century by using this effect [3]. In recent decades, these materials have drawn much attention due to their high gauge factor, exceptional mechanical properties, high thermal, large specific surface area, high electrical conductivities, low density, and remarkable piezoresistive properties [4]. The tensile strength and Young’s modulus of graphene and CNTs are 160 GPa and 1.0 TPa, respectively [5,6,7]. By reason of these unique properties, more attention is given to explore these materials as fillers [8,9,10] to improve the properties of other materials. Additionally, significant applications of CNTs and graphene can be found in newer areas, such as gas sensors, strain sensors, pressure sensors, energy storage, humidity sensors, temperature sensors, optical sensors, light emitting diodes, photovoltaic cells, chemical sensors, displacement sensors, solar cells, transistors, and other electronic equipment [3,11,12,13]. Pressure sensor is one of the key element in sensing technology that have been extensively studied in emerging industrial and electronics applications such as health monitoring devices, aviation, automotive, e-skin and touch screen devices [3,14,15].

Piezoresistive pressure sensors outperformed because of their fast response time, easier signal collection, simple operating principles, high flexibility, less complicated electronics requirements, low sensitivity to overloading, mechanical robustness, simple structure, and high sensitivity [8,16,17]. A literature survey shows that most of the scientists and researchers so far focused on utilizing CNT and graphene as fillers and to investigate its optical and electrical properties in the form of various types of sensors and other electronic devices. However, limited work has been extended to the piezoresistive properties of these materials. Thus far, to the best of our knowledge, the piezoresistive properties with various CNTs contents in CNTs-graphene composite samples under compression have not been reported earlier.

In this work, a novel and cost effective approach for the production of piezoresistive pressure samples is explored and tried to clarify the role of increase in the wt% of CNTs in CNT-graphene composite pellets. It is believed that this approach will further enhance the practical applications of CNTs, graphene, and its composites in the field of nanomaterial-based pressure sensors, actuators, and other electronic devices.

## 2. Materials, Sample Fabrication and Experimental Setup

### 2.1. Materials

Multi-walled carbon nanotubes (MWCNTs) and graphene nanopowders used in this study were commercially purchased from Sun Nanotech Co., Ltd., Nanchang, China. According to the supplier, the purity, range of length and outer diameter of multi-walled carbon nanotubes (MWCNTs) are >90%, 1–10 µm and 10–35 nm, respectively. The thickness range and area size of the graphene are 5–20 nm and 10 × 10 µm, respectively. The materials were used for the sample’s fabrication as received without further purification.

### 2.2. Samples Fabrication

Carbon nanotubes and graphene nanopowder were weighed by using analytical balance (Model: KERN ALS 220-4, weighing range (max): 220 g, readability (d): 0.1 mg, reproducibility: 0.2 mg, linearity: ±0.2 mg, warm-up time: 8 h, stabilization time (typical): 4 s, operating temperature: +18 °C to +30 °C, humidity of air: max. 80% (not condensing), weighing plate (stainless steel): 85 mm). Then both materials were carefully blended in a mortar and pestle to obtain a fine composite of the nanopowder. CNTs and graphene powder for all the samples were blended for the same time of 10 min. Then several samples were prepared from the blended materials and the resistances of samples of same amount, same size, same shape, and same thicknesses were compared with each other. They showed the same resistances which exhibits that the materials were mixed uniformly. We have fabricated all the samples in clean environment to reduce the effect of humidity and atmospheric contamination in it.

**Figure 1:**
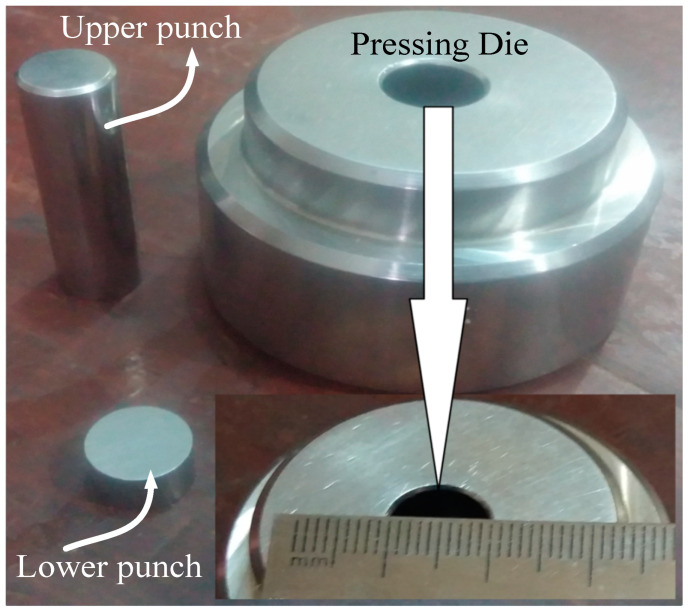
Pressing die with lower and upper punches.

The blend of CNTs and graphene nanopowder was loaded from the top into the fixed volume pressing die of 10 mm inner diameter. The top and bottom of the pressing die were closed with well fitted movable stainless steel punches as shown in the Figure 1.

**Figure 2:**
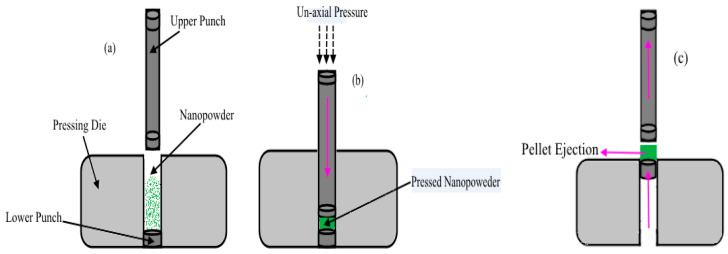
Samples fabrication process: (**a**) Die filling stage. (**b**) Pressed nanopowder. (**c**) Pellet ejection.

To make the pellets more durable, the blend of the material in pressing die was pressed by hydraulic press and then ejected from the die as shown in Figure 2a–c. Thickness and fabrication pressure of the prepared samples were 2 mm and 27,579.02 kNm^−2^, respectively.

**Figure 3:**
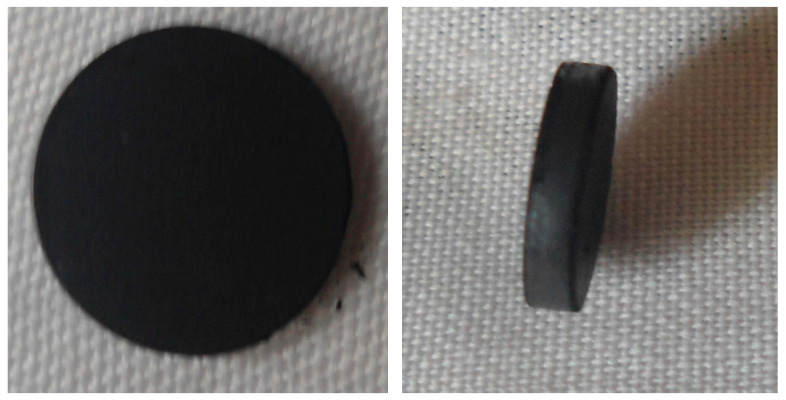
Photograph of one of the fabricated sample.

When the pellets’ conservation is not a requirement, commercially-available larger diameter dies could be used for the production of the samples. For the comparison purpose, four samples with CNTs weight percentages of 20, 40, 60, and 80 wt% were produced, respectively. All four pellets thus prepared according to the above procedure are shown in Figure 3.

For simplicity, the four samples with 20, 40, 60, and 80 wt% of CNTs are named as CNT 20, CNT 40, CNT 60 and CNT 80.

### 2.3. Setup and Measurements

**Figure 4:**
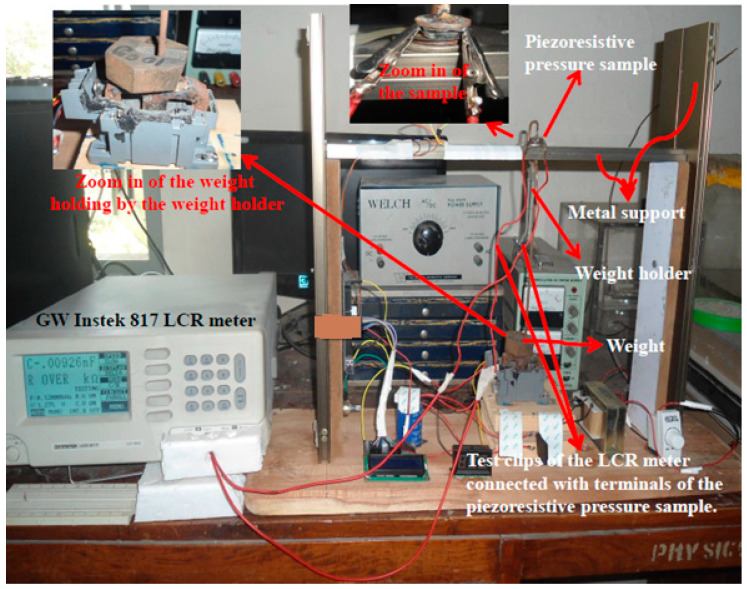
Experimental setup for the characterization of piezoresistive pressure samples.

For the characterization of the piezoresistive pressure samples, each sample was installed in the experimental setup as shown in the Figure 4.

**Figure 5:**
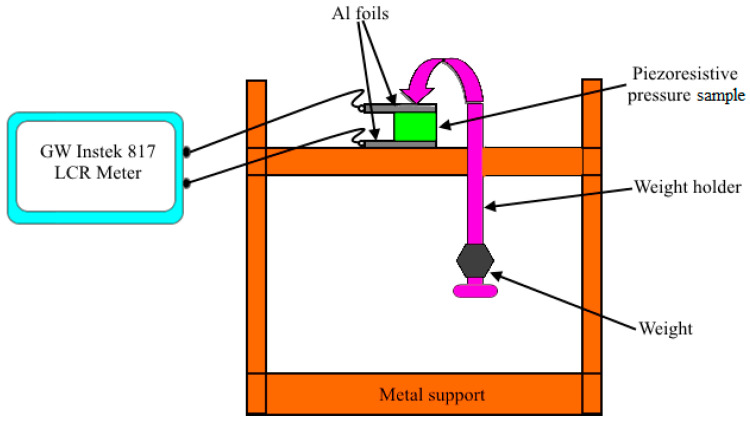
Corresponding schematic of the experimental setup of Figure 4 for the characterization of piezoresistive pressure samples with metallic support, weight holder, weight, sensor, and aluminum foils as terminals connected to a GW Instek 817 LCR meter.

The corresponding schematic concept of the experimental setup of Figure 4 is shown in Figure 5.

In Figure 5, the sample is placed on metal support. The upper curved end of the weight holder is placed on the sample. Then the weight, holding by the lower end of weight holder is increased from 0.1, 0.15, 0.2, 0.3, 0.4, 0.5, and 0.6 kg, which were converted to equivalent pressures of 6.2, 12.4, 18.7, 24.95, 37.4, 49.9, 62.4, and 74.8 kNm^−2^, respectively, by using the standard expression of P = W/A, where P is the pressure, W is the weight, and A is the cross-sectional area of the sample.

**Figure 6:**
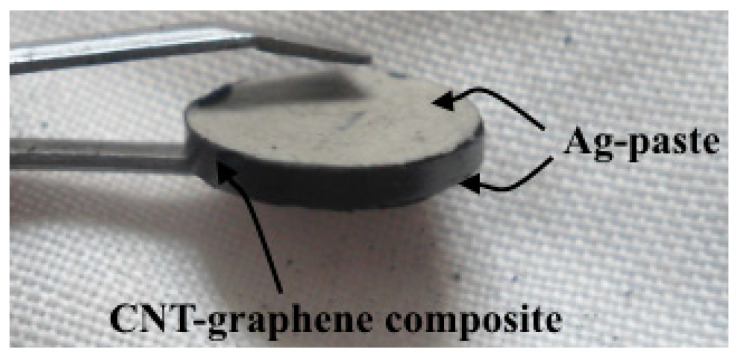
Carbon nanotube (CNT)- graphene composite sample with conductive silver (Ag) paste.

Both the surfaces of each prepared sample were coated with conductive silver paste to minimize the electrical contact resistance between the electrodes of the measurement equipment and surface of the samples as shown in Figure 6.

The aluminum (Al) foils are utilized (Figure 5) to act as terminals and to protect the sample from scratches as well. Silver paste are considered as a part of sample and hence not shown in the Figure 5. It can be seen from Figure 5 that the terminals (Al foils) of the piezoresistive pressure sample were connected with the test clips of the GW Instek 817 LCR meter. The specifications of the LCR meter are given in Table 1.

The initial resistance of each pressure sample at 0 kg was noted and then the value of the pressure was varied by the change in the weights held by the weight holder. Weights on the weight holder were increased as 0.05 (own weight of weight holder), 0.1, 0.15, 0.2, 0.3, 0.4, 0.5, and 0.6 kg while the corresponding pressure on the sensor was 6.2, 12.4, 18.7, 24.95, 37.4, 49.9, 62.4, and 74.8 kNm^−2^, respectively. The change in direct current (DC) resistance of the piezoresistive pressure sample with an increase in pressure was noted at normal temperature in ambient air from the display readings of a high-precision GW Instek 817 LCR meter. Special attention was directed to prevent any error in resistivity measurements. Five minutes were allowed prior to each resistivity measurement to avoid any transient effect in response of the sample.

## 3. Results and Discussion

### 3.1. Scanning Electron Microscopy

**Figure 7:**
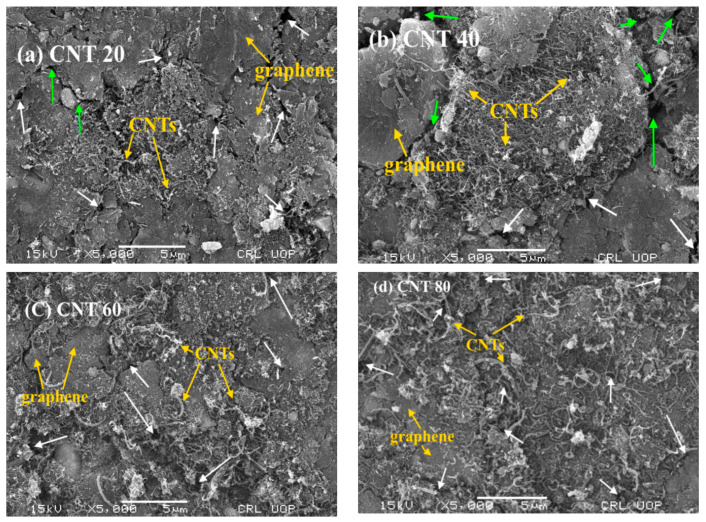
SEM images of CNT-graphene composite based piezoresistive pressure samples; (**a**) 20 wt% of CNTs, (**b**) 40 wt% of CNTs, (**c**) 60 wt% of CNTs, (**d**) 80 wt% of CNTs with cracks (green arrow) and voids/pores (white arrow).

The surface morphology of the piezoresistive pressure samples was examined by scanning electron microscope (SEM, model: JSM5910, energy: 30 KV, magnification (Max): 300,000×, resolution power (Max): 2.3 nm, manufacturer: JEOL, Tokyo, Japan). SEM images of piezoresistive pressure samples with different weight percentages 20, 40, 60, and 80 of CNTs (CNT 20, CNT40, CNT 60, and CNT 80) are shown in Figure 7a–d. Microscopic images of the all the samples have same magnification with a scale bar of 5 μm.

It can be observed from Figure 7a–d that CNTs and graphene are not uniformly distributed throughout the samples. Graphene sheets exhibit layered structures and mostly arranged in parallel planes orientation while most of the CNTs are in curved shapes and forest-like or entangled locally (yellow arrow). Some cracks (green arrow), voids and pores (white arrow) can be seen on the surfaces of the samples. No evidence in SEM images (Figure 7a–d) can be found to support that there were fractured carbon nanotubes and graphene layers on the surface of the pressed pellets. This shows the high flexibility and well suitability of these materials for the pressure sensors and other sensing devices. The surfaces of the samples (Figure 7a–d) are not fairly smooth. Some cracks (green arrow in Figure 7a,b), voids and pores (white arrow in Figure 7a–d) can be seen on the surfaces of the sensors. SEM images (Figure 7a–d) reveal that the size of pores and voids decrease as the CNTs contents increase in the composites. The size of the pores and voids in the sensors are smaller in the following order: CNT 80 (Figure 7a) < CNT 60 (Figure 7b) < CNT 40 (Figure 7c) < CNT 20 (Figure 7d). The reduction in porosity and voids efficiently increase the overall conductivity and, hence, decrease the resistance of the sample. Obviously, more CNT ropes can be seen in the SEM images (Figure 7a–d) as the weight percentage of CNTs increased from 20 to 80 in the composites.

### 3.2. Pressure vs. Resistance

**Figure 8:**
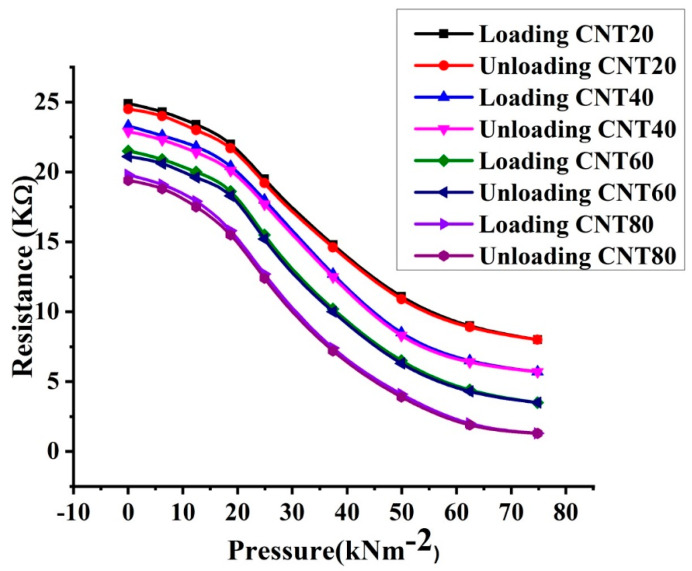
Resistance-pressure characteristics for CNT-graphene composite based piezoresistive pressure samples at loading and unloading with weight percentages 20, 40, 60, and 80 of CNTs.

Resistivity variation with loading and unloading of CNT-graphene composite based piezoresistive pressure samples for the weight percentages 20, 40, 60, and 80 of CNT is shown in Figure 8.

It can be seen from Figure 8 that all the four samples shows a decrease in the DC resistance as the pressure increase from 0 to 74.8 kNm^−2^. When the pressure was decreased from 74.8 kNm^−2^ back to zero, the curves did not overlap due to hysteresis effect in the composite materials. The existing pores and void spaces in the material act as a source of electrical resistance. The increase in pressure may crush the pores in the materials and facilitate the contacts between the neighboring particles that results increase in the conductivity and hence decrease the resistance of the samples. The characteristics behavior is almost identical for the composites fabricated with different wt% of CNTs. However, for the same pressure range, the resistivity of each sample decreases as the weight percentages of the CNTs increase in the composite. It can be observed from Figure 8 that the samples fabricated with small CNT contents have higher resistance than the samples fabricated with greater CNTs contents. The DC resistance of the composites with respect to wt% of CNTs is greater in the following order: Sample fabricated with 20 wt% of CNTs (CNT20) > sample fabricated with 40 wt% of CNTs (CNT40) > Sample fabricated with 60 wt% of CNTs (CNT60) > Sample fabricated with 80 wt% of CNTs (CNT80). The opposite trend can be observed with respect to wt% of graphene: Sample fabricated with 80 wt% of graphene (CNT20) > Sample fabricated with 60 wt% of graphene (CNT40) > Sample fabricated with 40 wt% of graphene (CNT60) > Sample fabricated with 20 wt% of graphene (CNT80). It can be attributed by two factors; (1) the electrical conductivity of CNTs is higher than the electrical conductivity of graphene nanopowder (2) the density of CNTs is lower than the density of graphene nanopowder [18,19,20]. As the amount of CNTs content in CNT-graphene composites increase from 20 to 80 wt%, the conductivity of the corresponding composite increase, which leads to a reduction in the resistance of the samples. The effect of the second factor (density) on the resistance-pressure relationship (Figure 8) of the composites is more significant, because the higher the density, the closer the particles and the smaller the void space between the particles in the materials. The higher density of graphene ensures that the total void space between graphene nanoparticles is smaller than the total void space between CNTs nanoparticles. The formation of conductive networks in the materials depends on the number of contacts between neighboring particles. The number of effective contacts increases under pressure due to the forced approach of the nanoparticles belongs to the neighboring aggregate [18,21,22]. Under the same external applied pressure, an increase in the resistivity with increase in graphene content in the CNT-graphene composites is observed in Figure 8. This is due to the better contacts and particles rearrangement in graphene as compared to CNTs nanopowder. The better contacts and particles rearrangement in graphene causes less densification and greater resistance of the composite under the same external applied pressure as shown in Figure 8.

The resistance-pressure relationships shown in Figure 8 for the samples CNT20, CNT40, CNT60, and CNT80 could be divided in three appreciable parts. In the low-pressure regime (0–12.4 kNm^−2^), there is no significant decrease in the resistance with increase in external uniaxial applied pressure. This behavior may be interpreted as the rearrangement of agglomerates present in the CNTs and graphene nanopowder. The resistance of the composites is drastically decreased under the pressure ranging from 12.4 to 60 kNm^−2^. This may be attributed by the densification effect occurs in the press tablets under the external uniaxial applied pressure. The densification and squeezing effects causes additional deformation in the structure of the press pellets by decreasing the interfacial distance between the neighboring particles and increasing the concentration of the charge carriers in the materials. The increase in charge carrier’s concentration may fill the localized energy states present between the HOMO-LUMO levels which may lead to increase the electrical conductivity and, hence, decrease in the resistance of the samples. When the pressure comes to a higher region (60–74.8 kNm^−2^), the resistance-pressure relationships are flatten out because the percolation threshold is reached and no more effective production of the conductive networks occurs within the sample elements. The resistance value of the sensor elements not tends to zero exactly even at a higher pressure. This can be interpreted as follows: Beyond a certain value of the pressure, called percolation threshold (60 kNm^−2^), the total pores and void spaces in CNTs-graphene composites may not be completely eliminated from the volume of the pellets.

The resistance, ‘R’ of the sensor elements can be calculated by Equation (1) [23,24]:

(1) $$R=\frac{d}{\sigma A}\;$$

where σ is the conductivity, d is the thickness, and A is the cross-sectional area of the sensor element.

The overall conductivity of the pressed tablets contributed to the contact resistances between the neighboring particles of carbonaceous material. The intrinsic resistances are extensively distributed throughout the sample’s volume due to the different particle sizes in the materials. Under the external uniaxial applied pressure, the contact areas between the grains in carbonaceous materials may be different due to the irregular deformation of grains across the whole sample. It is very difficult to find the actual geometry of the conductive paths within the materials. The contacts made by the neighboring particles with each other are due to the touching points instead of well-defined surfaces. Therefore, the number of conductive networks throughout the volume of the sensor element is unpredictable. Additionally, the distribution of forces within the compact carbonaceous materials is extremely inhomogeneous [25]. The problem becomes even more complicated if the friction forces of material particles against the walls of the sample holder are considered. Many more difficulties, such as crumbling of grains under the external applied pressure, could be taken into account. However, a list of the few barriers mentioned above is enough to say that the resistivity- and conductivity-related issues of the carbonaceous materials cannot be resolved exactly. Some assumptions, approximations, and simplifications are required to deal with such complicated disordered systems. Percolation theory is one of the suitable tools to investigate different phenomena in such randomly-distributed systems. Numerous articles related to the applications of percolation theory in randomly-distributed and heterogeneous systems can be found in the existing literature [26]. According to percolation theory, the average conductivity of a single component (CNT-graphene composite in this case) can be calculated by Equation (2) [23]:

(2) $$\sigma =\frac{1}{\mathrm{LZ}}\;$$

where Z is the resistance of the path with lower average resistance and L is the concentration of the particle in multicrystalline disordered carbonaceous materials. As the external uniaxial pressure increases, the density of the sample elements increases which increase the nanoparticle concentration (L). The increase in L causes a reduction in Z. Therefore, the electrical conductivity increases and hence a decrease in the resistance of the composites is observed in resistance-pressure relationships as shown in Figure 8.

### 3.3. Experimental vs. SIMULATION

The response of a sample can be approximated either by linear, exponential, or polynomial functional approximation [27]. Since the characteristics of all the four composites have identical shapes, therefore, one of the above mentioned functional approximations can be applied to all the CNT-graphene composite based piezoresistive pressure samples. In our case, the third-order polynomial functional approximation (Equation (3)) is used to best fit the experimental data, which is given as:

Y = C_0_ + C_1 ×_^1^ + C_2 ×_^2^ + C_3 ×_^3^ (3)

For the resistance-pressure relationships, Equation (3) can be written as:

R = C_0_ + C_1_P^1^ + C_2_P^2^ + C_3_P^3^ (4)

where P is the external uniaxial applied pressure, R is the DC resistance of the sample, C_0_ is the intercept that represents the DC resistance of the samples at zero external applied pressure, and C_1_, C_2_, and C_3_ are the fitting parameters.

**Figure 9:**
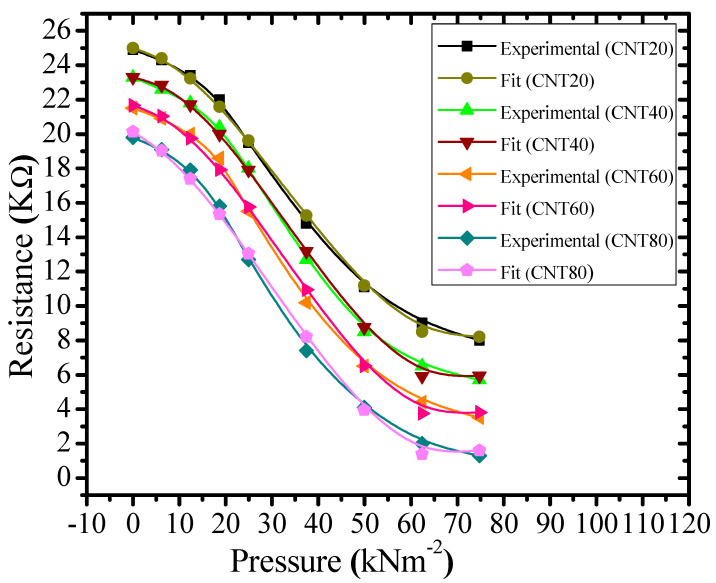
Resistance-pressure characteristics of experimental data (Figure 8) and fitted curves (Equation (4)) for CNT-graphene composite-based piezoresistive pressure samples with weight percentages of 20%, 40%, 60%, and 80% of CNTs.

The experimental resistance-pressure relationships (Figure 8) and simulated results (Equation (4)) for the composites with 20, 40, 60, and 80 wt% of CNTs are shown in the Figure 9.

The values of C2 and C_3_ for each composite are shown in Table 2.

Deviations of the experimental data (Figure 8) from the fitted results (Equation (4)) are 0.08%, 0.15%, 0.5%, and 2.4% for the samples CNT20, CNT40, CNT60, and CNT80, respectively. Experimental data and fitted results exhibit excellent agreement with each other.

The amount of deviation of all the experimental characteristics from the fitted curves is calculated by using Equation (5) [28]:

(5) $$\%\;\mathrm{Deviation}=\frac{\mathrm{Theoretical}\;\mathrm{value}\;-\;\mathrm{Experimental}\;\mathrm{value}}{\mathrm{Theoretical}\;\mathrm{value}}\times 100\;$$

## 4. Conclusions

Multi-walled carbon nanotubes (MWCNTs)-graphene composite based piezoresistive pressure samples in the form of pellets were fabricated by mortar and pestle/hydraulic press technique. For comparison purpose, four samples with CNTs with weight percentages of 20%, 40%, 60%, and 80% were produced, respectively. Under the same external uniaxial applied pressure, the resistivity of the first four samples decreased as the weight percentage of the CNTs increased in the composite.

Percolation theory was invoked to provide a qualitative description of a potential conduction mechanism in the fabricated composite samples. The resistance-pressure characteristics were fitted and compared with experimental data. Deviations of the experimental data from the fitted results were 0.08%, 0.15%, 0.5%, and 2.4% for the samples with CNTs weight percentages of 20%, 40%, 60%, and 80%, respectively. For all the samples, experimental data shows excellent agreement with the fitted curves.

## Figures and Tables

**Table 1:** Specifications of the GW Instek 817 LCR meter.

Test Frequency	Basic Accuracy	Test Speed	Test Signal Levels	Measurement Range	Quality 75 Factor	Dissipation Factor
12 Hz–10 kHz	0.05%	68 ms	5 mV–1.275 Vrms	0.00001 Ω–99,999 kΩ	0.0001–9999	0.0001–9999

**Table 2:** Values of intercept C_O_ and the fitting parameters C_1_, C_2_, and C_3._

Composite	C_O_ (kN^−2^m^2^)	C_1_ (kN^−2^m^2^)	C_2_ (kN^−2^m^2^)	C_3_ (kN^−2^m^2^)
CNT20	25	−0.043	−0.009	9.07 × 10^−5^
CNT40	23.3	−0.006	−0.012	1.08 × 10^−4^
CNT60	21.6	−0.041	−0.011	1.05 × 10^−4^
CNT80	20.2	−0.129	−0.008	9.34 × 10^−5^
